# Flow cytometric assessment of DNA double-strand break and repair kinetics in prediction of intrinsic radiosensitivity 

**DOI:** 10.22038/IJBMS.2022.65178.14350

**Published:** 2022-09

**Authors:** Mohammad-Taghi Bahreyni-Toossi, Mahdieh Dayyani, Mahmoud Mahmoudi, Hosein Azimian

**Affiliations:** 1 Medical Physics Research Center, Mashhad University of Medical Sciences, Mashhad, Iran; 2 Reza Radiotherapy Oncology Center, Mashhad, Iran; 3 Immunology Research Center, Bu-Ali Research Institute, Faculty of Medicine, Mashhad University of Medical Sciences, Mashhad, Iran

**Keywords:** Breast cancer, DNA damage, Double-strand break, Radiosensitivity, Radiotherapy

## Abstract

**Objective(s):**

To enhance the efficiency of radiotherapy (RT), implementation of individual-based treatment is essential. In this way, determining individual intrinsic radiosensitivity (IRS) can be useful to achieve minimal adverse effects of RT. The present study aimed to identify IRS of breast cancer (BC) patients through determination of radiation-induced DNA double-strand breaks (DSBs), repair kinetics, and acute normal tissue complications induced by RT.

**Materials and Methods:**

DSBs induction and its repair kinetics in 50 BC patients’ lymphocytes were analyzed by flow cytometric analysis of H2AX Ser-139 phosphorylation at 30 min, 3 and 24 hr after in vitro irradiation. In vivo skin dosimetry was done by GAFChromic films and acute skin toxicity was scored by radiation oncologists according to the criteria of Radiation Therapy and Oncology Group (RTOG) in all patients with similar prescribed treatment.

**Results:**

The average surface dose for patients ranged from 0.92 to 1.9 Gy and correlation analysis showed no significant relationship with weekly acute skin reactions. Formation of γH2AX after 30 min, slope of dose-response curve and repair kinetics of DSBs after 3 and 24 hr (intrinsic radiosensitivity) were significantly correlated with the RTOG scores following irradiation (clinical radiosensitivity) (r=0.48 and *P-value<*0.0001, r=0.72 and *P-value<*0.0001, r=0.48 and *P-value<*0.001, and finally r=0.53 and *P-value<*0.001, respectively; (using Pearson’s correlation test).

**Conclusion:**

Flow cytometric analysis of DNA DSBs by γH2AX measurement has the potential to be developed into a clinical predictor for identifying the overreactor patients prior to RT. Our result suggests that the slope-related quantity based on the linear pattern of the dose-response curve has the merit to predict overreactor patients with a sensitivity of 89% and a specificity of 94%.

## Introduction

Radiotherapy (RT) is one of the common cancer treatment modalities, but the responses to RT vary considerably between individual patients (1). Evidence suggests that healthy tissues adjacent to the tumor exposed to radiation during RT are more prone to acute or permanent damage (2). Curative RT-induced acute and chronic toxicities are substantial limitations for efﬁcient RT. In a way, acute toxicities can result in dose reductions, interruptions of treatment, and even discontinuation of RT, and chronic toxicities worsen the quality of life (3). However, radiation-induced toxicity severity depends on multiple conditions, resulting from a complex interaction between tumor microenvironment, patient biology, and treatment-related factors(4). Generally, high-grade toxicity in 16 to 23% of patients during or shortly after completing the RT course has been reported in several studies (5-8). Undoubtedly, these normal tissue side effects are caused by something other than tumor characteristics and treatment plans. Understanding the patient-dependent parameters could be the missing link that helps to predict the outcome of RT (9). Intrinsic radiosensitivity (IRS) is one of the most important biological factors related to the patient that determines the probability of normal tissue complication and a successful tumor cure (10). Individual assessment of IRS holds the hope that cancer therapy moves towards personalized medicine (11). Some promising approaches have been investigated recently, such as the surviving fraction at 2Gy (SF2)(12), DNA repair capacity (13), gene expression levels(14), and miRNA expression (15). Although SF2 as the conventional indicator to determine IRS has also been confirmed in *ex vivo* studies, plating efficacy of isolated cells of tissue biopsy is very low and this assay will be very time-consuming for clinical uses (16). For these reasons, alternative methods are needed to be developed to measure IRS. Recently, repair capacity of DNA double-stranded breaks (DSBs) in the classification of cell lines radiosensitivity by γH2AX assay and validation by SF2 was shown (17). 

This study evaluates the correlation of the radiation-induced normal tissue toxicities, repair kinetics of phosphorylation of histone variant H2A.X (γH2AX) in peripheral blood mononuclear cells (PBMCs), and the skin dose received by individual breast cancer (BC) patients.

## Materials and Methods

***Subjects***

The analysis of PBMCs from fifty unselected BC patients prospectively involved in the study. Collection of the blood samples were approved by the Ethics Committee of Mashhad University of Medical Sciences (approval code: IR.MUMS.REC.1394.59) and written informed consent was obtained from all patients prior to blood collection. 

***Radiotherapy techniques***

RT treatment of BC patients was performed by a 3D conformal technique (3D-CRT) using linear accelerator (Siemens, Concord, CA, USA) at a dose rate of 2-3 Gy/min for energies 6 and 15 MV, respectively. A 5-mm-thick tissue-equivalent bolus was used to supply the dose distribution uniformity on the chest wall surface for 15 MV energy tangential fields. Conventional fractionation (2 Gy/fraction/day, five days/ week) with the total dose 50–60 Gy was applied for all participating patients administered tangential whole breast RT with virtual wedge.

***Clinical radiosensitivity***

Radiation-induced normal tissue toxicities as clinical radiosensitivity indicators were used in this study. The development of acute skin reactions to RT in the radiation field were controlled weekly according to Radiation Therapy and Oncology Group (RTOG) score (18). 

***In vivo skin dosimetry***

In order to investigate the possibility of association between absorbed dose and acute skin reactions, *in vivo* dosimetry was carried out by using radiochromic EBT-3 films (International Specialty Products, USA) as described previously (19). Five cm^2^ pieces of films were placed on the medial, lateral, and center of the treatment field of patients’ chest wall. The net optical density of each piece of the film was analyzed in the red channel, which has the optimum response at doses up to 10 Gy. The program written in MATLAB software (R2015b) for analyzing films is presented in Appendix A of the paper.

***Lymphocyte cultures and in vitro irradiation***

All *in vitro* analysis for IRS estimation was carried out on patients’ samples collected before treatment. PBMCs were isolated by density centrifugation on Ficoll-Hypaque® gradients (Cedarlane Laboratories, Canada). Isolated PBMCs were re-suspended in RPMI 1640 (GIBCO, Germany) supplemented with 10% fetal bovine serum (FBS, Biosera, France), 100 Iu/ml penicillin, and 100 µg/ml streptomycin at a cell density of 10^6^ cells/ml in a T-25 flask and incubated at 37 ^°^C with 5% CO_2_. *In vitro*  irradiation was performed by 6 MV X-rays linear accelerator (Siemens, Concord, CA, USA) at a dose rate of 2 Gy/min to deliver 1 and 2 Gy. Control cells were treated similarly, but without radiation dose. Immediately, irradiated and control samples were incubated at mentioned conditions for 30 min, 3 hr, and 24 hr. 

***DNA double-strand breaks and repair kinetics analysis***

Phosphorylation of histone H2A.X (γH2AX) were measured at different time points after *in vitro* irradiation for all patients to evaluate individually DNA double-strand breaks (DSBs) and repair capacity. Flow cytometry analysis of γH2AX in PBMCs was performed with the H2AX phosphorylation detection kit (Millipore, USA). Briefly, cells were washed twice and then fixed with formaldehyde/methanol solution (Catalog #12-487, Millipore, USA) and finally incubated on ice. Fixed cells were washed to remove the fixation buffer. Then, 50 µl of the 1X permeabilization solution (Catalog #20-259, Millipore, USA) and 3.5 µl of anti-phosphorylated Histone H2AX (Ser139), FITC-conjugate (Catalog #16-202, Millipore, USA) were added to the cellular pellet. Finally, cells were washed with 100 µl of PBS/saponin solution (Catalog #20-258, Millipore, USA). All samples were re-suspended in PBS containing 40 µg/ml propidium iodide (PI). Consequently, fluorescence intensities were measured with a two-color FACSCalibur™ flow cytometer (BD Biosciences), and data were analyzed using FlowJO (TreeStar, CA, USA).

***Statistical analysis***

All statistical analyses were performed using GraphPad Prism, version 8.02. Data were collected from three independent experiments and indicated the mean ± standard deviation (SD). Statistical analyses were performed by one-way analysis of variance (ANOVA). The Pearson correlation coefficient was used to assess the correlation between the variables.

The Kolmogorov-Smirnov was used to assess the goodness of fit of a Gaussian distribution. The thresholds for predicting overreactor patients were obtained using Gaussian distribution. The bounds of this range were evaluated by fitting the 68^th^ and 95^th^ centiles of the Gaussian distribution. Finally, we produced a ROC curve by plotting the sensitivity and 1-specificity for various cutoff points to predict acute skin toxicity.

## Results

All patients’ tumor stages (according to the TNM criteria of BC), acute skin reactions, RT plan details, skin surface doses, and IRS index were recorded (Appendix B) and summarized in Table 1.

***Surface dose versus acute reactions of the skin***

In order to assess treatment-related factors and their possible effects on radiation dermatitis, skin surface dose was determined for all patients in three areas, as previously described. The average surface dose for patients ranged from 0.92 to 1.9 Gy (detailed data are given in Appendix B). Pearson’s correlation analysis showed no significant interrelationship between surface dose and weekly acute skin reactions (Pearson correlation coefficient r=0.09).

***DNA double-strand breaks versus acute reactions of the skin***

**Figure 1:**
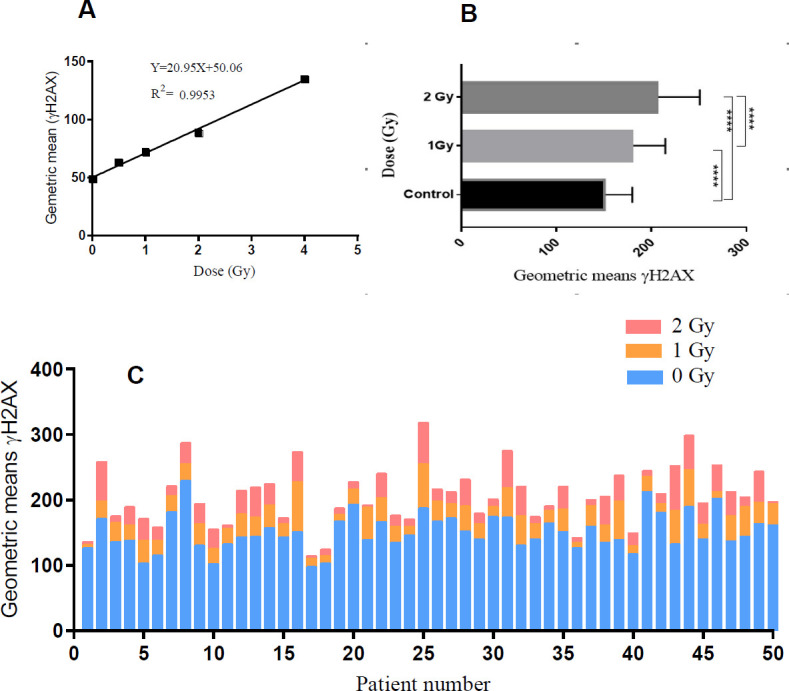
The dose-response curve for geometric mean of γH2AX

Firstly, as shown in Figure 1A linear dose-response relationship was generally observed for the geometric mean of γH2AX at 30 min after radiation (R^2^=0.99). Also, mean values of γH2AX geometric mean for all patients (Figure 1B) showed significant differences between doses (*P*-value< 0.0001). The distribution of γH2AX geometric mean based on Kolmogorov-Smirnov was normal (the adjusted R-square of the Gaussian function was 0.91). However, as shown in Figure 1C each patient had a unique dose-response pattern.DSBs induced by radiation was calculated using the formula: 

DSBs=[Geometric mean of γH2AX_(30 min post-irradiation) _- Geometric mean of γH2AX_(control)_].

**Figure 2:**
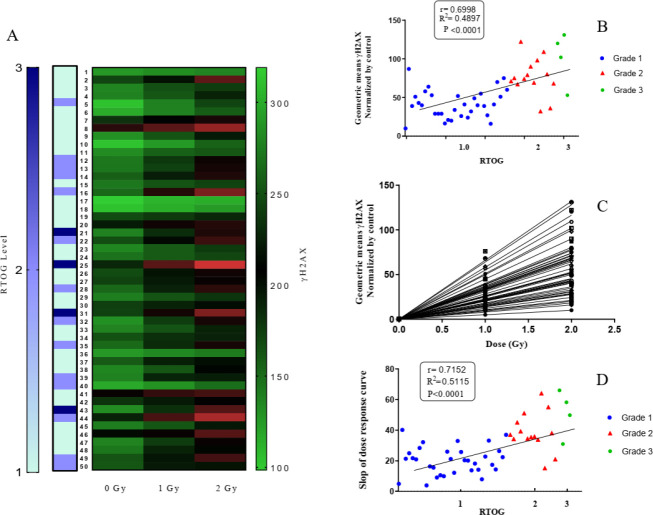
(A) Heat map of the γH2AX amount and RTOG levels for each BC patient in *in vitro* irradiated lymphocytes; (B) scatter plot of γH2AX function according to RTOG levels for 2 Gy; (C) dose-response curve by patients after normalization with the control, and finally; (D) slope of dose-response curve for patients as a function of RTOG scales

Heat map of DNA DSB induced by 0-2 Gy and RTOG scales for fifty BC patients who participated in the study is represented in Figure 2A. The utilization of heat map correlation between RTOG scale and γH2AX amount for patients 25 and 31 is interesting. Also, scatter plot of normalized geometric means of γH2AX for 2 Gy is represented in Figure 2B. 

As shown in Figure 2C dose-response curve provides a slope-related quantity based on the linear pattern of changes for each patient. Interestingly, the slope of the curve is significantly correlated with RTOG scales for each patient (*P*-value<0.0001, R^2^=0.51) (Figure 2D).

***Residual DSBs and clinical side effects***

Residual DSBs is the amount of unrepaired damage at subsequent times of γH2AX assessment which is calculated as follows:

%Residual DSBs _=_ [γH2AX_ (3 or 24 hr__ (_-γH2AX_ (control)_]/ [γH2AX_ (30 min__ (_-γH2AX_ (control)_]

**Figure 3:**
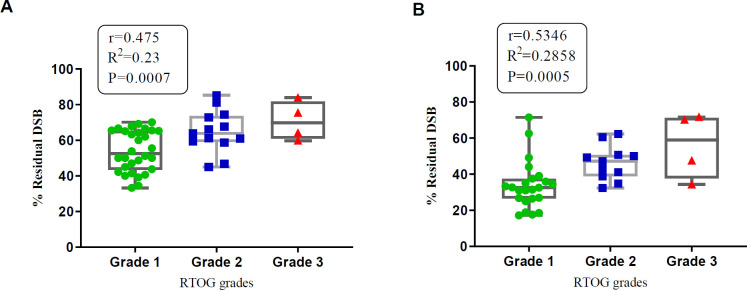
Distribution of residual DNA double-strand break (DSBs) in *in vitro* irradiated lymphocytes of 50 breast cancer patients. (A) Scatter plot of residual DSBs at 3 hr after 2 Gy irradiation to all patient samples with RTOG grades of acute skin reaction. (B) Scatter plot of residual DSBs at 24 hr after 2 Gy irradiation to all patient samples with RTOG grades of acute skin reactions

As shown in Figure 3 (A-B) acute skin reactions are significantly correlated with residual DSBs at 3 and 24 hr post-irradiation (r=0.475, *P*-value<0.001) and (r=0.53, *P*-value<0.001), respectively. The normal distribution assumption of residual DSBs based on Kolmogorov-Smirnov was confirmed at 3 hr (R-square= 0.91).

***Predictive value analysis***

So far, we observed that the levels of γH2AX at different time points are significantly different between the groups of BC patients. However, the predictive performance of the *in vitro* radiosensitivity indexes should be checked by statistical analysis. Based on the Gaussian function for a normal distribution, 68% of the population are within +/- SD of the mean, and 95% are within +/- two SD. When taking a binary predictive assay based on the normal distribution of the “Geometric mean of γH2AX (30 min)”, “residual DSBs at 3 hr”, and “slope of dose-response curve” the optimal cut-off values are introduced. Based on these thresholds’ values (Table 2), the slope of the patients’ dose-response curve had the best predictive performance (specificity, sensitivity, accuracy, negative predictive value (NPV), positive predictive value (PPV): 100%, 44.4%, 80%, 76%, and 44%, respectively).

**Figure 4:**
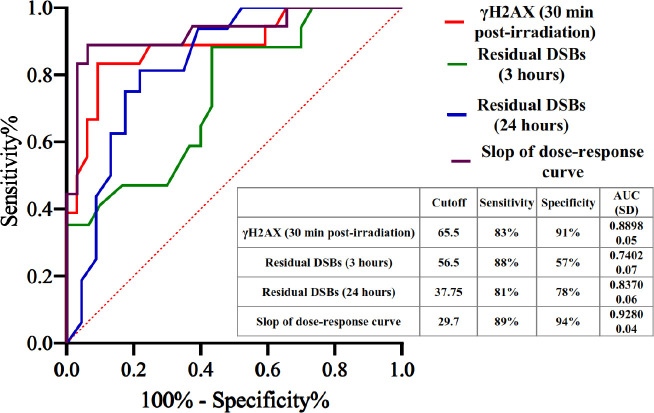
Validation of IRS estimation of grade 2–3 acute skin toxicity in BC patients. ROC curve of the γH2AX levels 30-min post-*in vitro* irradiation, residual DSBs (at 3 hr), residual DSBs (at 24 hr), and slope of dose-response curve are shown along with cut-off values, sensitivity, specificity, and AUC

In order to determine the predictive value of γH2AX for estimate of grade 2-3 acute skin toxicity, receiver operating characteristic (ROC) curve analysis was performed. The cut-off values were developed using the experimental obtained data of the level of γH2AX induced 30-min post-*in vitro* irradiation, residual DSBs (at 3 hr), residual DSBs (at 24 hr), and slope of dose-response curve (Figure 4).

## Discussion

The purpose of the present study was to determine the relationship between IRS and acute normal tissue side effects of fifty BC patients who underwent whole breast conventional RT. Radiation-induced side effects in the skin of each patient have been used as an individual clinical radiosensitivity index. The results are discussed below in three sub-sections: the first on the radiosensitivity background, the second on the result of radiation sensitivity in this research and comparison with other studies, and the third expression of sensitivity, specificity, and accuracy of the results for predicting patient IRS.

It is worth noting that RT remains an essential curative treatment modality due to fewer severe side effects and local invasion compared with surgery and chemotherapy. Unfortunately, mainly due to the heterogeneity of the patients, the tumor response rate and normal tissue reactions can vary considerably. Currently, most patient management decisions are based on crude clinical parameters related to primary tumor mass with little appreciation of the underlying tumor biology (20). Significant advances toward precise and personalized RT have been primarily achieved by advances in physical properties for treatment planning and dose delivery. In contrast, understanding the biological variables based on differences between patients that define IRS have not achieved the same success. Consequently, RT is prescribed and delivered without considering the patient differences in IRS after more than 100 years from the beginning (21).

Therapeutic effects of RT refer to its ability to cause lethal damage in tumor cells. On the other hand, RT side effects refer to sub-lethal damages in healthy tissues, which can be repaired by several pathways in normal cells. Defect in the DNA repair pathways is proposed as a radiosensitizing factor in healthy tissue (22). To the best of our understanding, the repair capacity of DNA DSBs is different in the sensitive, moderate, and resistant cells. Bahreyni *et al*. recently showed that classifying cell lines into three groups of radiosensitivity is possible by γH2AX assay (17). Several studies have been done to determine the potential of γH2AX in predicting treatment outcomes that are promising for clinical applications (23, 24). 

We have quantified the breast skin acute reactions to RT according to the RTOG score. Statistical analysis revealed that 36% of the patients suffered grade ≥2 injuries, and 64% were placed in grades 0 and 1. Pearson correlation analysis showed that these findings were not significantly related to the surface dose measured by GAFChromic EBT-3 films. The Heat map presentation of the γH2AX amount and RTOG levels in Figure 3.A indicates that among the four patients with higher skin acute reactions (RTOG =3), two patients showed more severe DNA DSBs (patients 25 and 31). Although such a coincidence could not be found for patients 21 and 43, these results conclude that a relationship between IRS measured as DNA DSBs in PBMCs and clinical side effects exists. Consequently, as shown in Figure 2.B. γH2AX levels induced by 2 Gy in the patients’ PBMCs after 30 min are correlated with RTOG levels. 

On the other hand, the dose-response curve for each patient is unique and seems to be related to IRS (Figure 2.C). In order to validate this assumption, slope-related quantity based on the linear pattern of changes was determined. The comparison with acute skin side effects in Figure 2.D shows significant correlation for all patients.

Finally, the repair kinetics after irradiation were evaluated after 3 hr, and residual γH2AX 24 hr’ post-irradiation was measured as the unrepaired DNA DSBs. We have demonstrated a significant correlation between persistence of the γH2AX post-irradiation and normal tissue toxicity.

Determination of the predictive value of γH2AX for grade 2–3 acute skin toxicity estimation, as shown in Figure 4, was performed by ROC curve analysis. A threshold of 29.7 of the dose-response curve slope displayed the most predictive power so that the area under the curve (AUC), sensitivity, and specificity in predicting grade ≥2 injuries were 0.928, 89%, and 94%, respectively. 

In accordance with the present study, Djuzenova *et al*. also observed significantly higher microscopic fluorescent γH2AX foci and lengthy disappearance in radiosensitive BC patients compared with patients with grade 0-1 skin reactions (25). In 2020, researchers reported higher levels of γH2AX expression in surgically resected specimens of radioresistance colorectal cancer patients (26). The potential of residual foci at 24 hr to distinguish individuals with late normal tissue reactions compared with normal patients has been demonstrated by Chua *et al*. (27). Their study was retrospective and compared a group of 8 overreactors with eight normal patients. Contrary to the present results, a study did not find any correlation between normal tissue response to RT and early levels of DNA DSBs by microscopic γH2AX assay in BC patients. However, their findings that showed significantly more serious residual damage at 6 hr in over responders versus normal patients are consistent with our result(28). Several more studies using microscopic immunofluorescence have reported a relationship between residual γH2AX foci and normal tissue toxicities caused by IR (29, 30). 

Although the microscopic immunofluorescence method has some potential, however, it has been shown at this stage, the visual scoring is time-consuming and potentially prone to errors and inconsistency (31). In 2015, a study described the microscopic method problems and estimated the IRS using γH2AX western blot analysis (32). Recently, Mahmoud *et al*. have revealed ELISA technique can measure γH2AX in the blood plasma of BC patients as a biomarker of radiosensitivity (33).

On the other hand, flow cytometry is presented as a method that can rapidly detect γH2AX fluorescence intensity in the large cell population (34, 35). In recent years, only a few studies have evaluated a similar correlation between γH2AX levels and acute skin reactions in cancer patients using flow cytometric γH2AX assay. Bourton *et al*. indicated defects in DNA repair mechanisms for 12 patients who had RTOG grades 3 and 4 after RT. They used flow cytometric analysis of γH2AX in the PBMCs samples that were taken after RT of different cancer type patients (36). However, a study in 2013 could not find any significant differences between the ten radiosensitive prostate cancer patients and twenty control samples (37). Differences in the results may be due to retrospective type of research and late effects that have been evaluated.

**Table 1:** Clinical details for all BC patients who participated in this study

Patients details	Value
No. of Patients	50
Age	29-79
Mean Median 49 ≤ 49 >	50.5 49 24 26
Tumor staging	
T_1_ T_2_ T_3_	2 29 19
RTOG Skin reaction grade	
0 1 2 3 4	0 32 14 4 0
Surface dose (mean±SD)	
Central Medial Lateral	1.36±0.28 1.31±0.35 1.28±0.32

A: For one patient; B: For mean values of all patients±standard deviation. **** Represent *P-value<*0.0001; C: For each patient separately

**Table 2:** Predictive performance of the radiosensitivity indexes introduced in this study

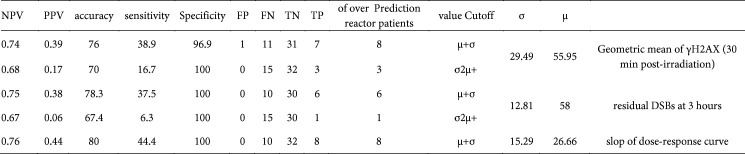

μ: mean; σ: standard deviation; TP: true positive; TN: true negative; FN: false negative; FP: false positive; PPV: positive predictive value; NPV: negative predictive value

## Conclusion

Briefly, the present study results suggest that γH2AX flow cytometric analysis provided four important markers to prospectively assess the IRS. Higher γH2AX levels at 30 min were linked with high grades of normal tissue toxicity. Also, high residual γH2AX at 3 and 24 hr after irradiation, which indicates poor DNA DSBs repair activity, can predict poor radiation tolerance. Our results suggest that the slope-related quantity based on the linear pattern of dose-response curve has worthiness to predict overreactor patients with the higher predictive value, including AUC, sensitivity, and specificity. The results of the present study indicate that the γH2AX flow cytometric analysis of DNA DSBs and repair kinetics could become a clinically promising predictive biomarker to identify radiosensitive BC patients prospectively.

## Authors’ Contributions

MTBT, MD, and HA conceived the study. MD performed patient screening. All experiments and data analysis were performed by MM, MD, and HA. HA wrote the manuscript, all authors have read and commented on the manuscript.

## Conflicts of Interest

The authors declare that no conflict of interest exists. 
